# Increased epigenetic diversity and transient epigenetic memory in response to salinity stress in *Thlaspi arvense*

**DOI:** 10.1002/ece3.6795

**Published:** 2020-09-20

**Authors:** Yupeng Geng, Na Chang, Yuewan Zhao, Xiaoying Qin, Shugang Lu, M. James C. Crabbe, Yabin Guan, Ticao Zhang

**Affiliations:** ^1^ Institute of Ecology and Geobotany School of Ecology and Environmental Sciences Yunnan University Kunming China; ^2^ School of Life Sciences Yunnan University Kunming China; ^3^ Wolfson College Oxford University UK; ^4^ Institute of Biomedical and Environmental Science & Technology University of Bedfordshire Luton UK; ^5^ School of Life Science Shanxi University Taiyuan China; ^6^ College of Chinese Material Medica Yunnan University of Chinese Medicine Kunming China

**Keywords:** adaptive evolution, DNA methylation, epigenetic diversity, field pennycress, salinity stress

## Abstract

Epigenetic diversity could play an important role in adaptive evolution of organisms, especially for plant species occurring in new and stressful environments. *Thlaspi arvense* (field pennycress), a valuable oilseed crop, is widespread in temperate regions of the northern hemisphere. In this study, we investigated the effect of salinity stress on the epigenetic variation of DNA methylation and epigenetic stress memory in pennycress using methylation‐sensitive amplification polymorphism (MSAP) markers. We examined how the status of DNA methylation changes across individuals in response to salinity stress and whether such an effect of maternal stress could be transferred to offspring for one or two generations in nonstressed environments. Our results based on 306 epiloci indicated no consistent change of DNA methylation status in specific epiloci across individuals within the same conditions. In contrast, we found that the epigenetic diversity at population level increased significantly in response to the stimulation of salinity stress; and this “stimulation effect” could be transferred partially in the form of stress memory to at least two generations of offspring in nonstressed environments. In addition, we observed a parallel change in functionally important traits, that is, phenotypic variation was significantly higher in plants grown under salinity stress compared with those of control groups. Taken together, our results provide novel clues for the increased spontaneous epimutation rate in response to stress in plants, of potential adaptive significance.

## Graphical abstract

Epigenetic diversity might play an important role in ecology and evolution. *Thlaspi arvense* (field pennycress) is an emerging model oilseed crop and widespread in temperate regions of the northern hemisphere. Our results provide novel clues for the increased spontaneous epimutation rate in response to stress in plants.

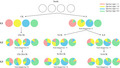

## 1 INTRODUCTION

Epigenetic modifications, such as DNA methylation, are chemical modifications of chromatin that can cause a change in gene expression, without altering the DNA sequence (Deichmann, 2016). Recent studies suggested that there are enormous epigenetic variations in natural populations (Richards et al., 2017). At least some of them might have significant effects on phenotypic variation and are heritable across generations (Li et al., 2020; Rambani et al., 2020; Verhulst et al., 2016; Wilschut, Oplaat, Snoek, Kirschner, & Verhoeven, 2016). Thus, epigenetic variations can contribute to epigenetic diversity, which is considered as the total epigenetic variation within a population or within a species, and might play an important role in ecology and evolution (Hirsch, Baumberger, & Grossniklaus, 2012). Specifically, epigenetic diversity can contribute to a distinct evolutionary pathway for organisms to adapt to environmental changes. In other words, rapid evolution might occur even in populations with limited genetic variation, as in the case of asexual lineage or the epigenetic recombinant inbred lines (epiRILs) of selfing plant species (Cortijo et al., 2014; Wilschut et al., 2016; Zhang, Latzel, Fischer, & Bossdorf, 2018).

DNA methylation variants can arise from spontaneous epimutations, as a consequence of environmental perturbations, or be determined by genetic variations of DNA sequence (Jones, 2012). In particular, DNA methylation status can be altered due to environmental stress, leading to the up‐ or down‐regulation of some genes. These genes are often involved in the molecular machinery responsible for plant stress responses. In that case, the epigenetic changes are often rapid, reversible, and consistent across replicative individuals (Lea, Altmann, Alberts, & Tung, 2016; Mirouze & Vitte, 2014; Wilschut et al., 2016). The induced epigenetic changes might be associated with phenotypic plasticity or physiological acclimation to environmental stress (Childebayeva et al., 2019; Cortijo et al., 2014; Zhang et al., 2018). Many studies have investigated the adaptive significance of epigenetic changes with various environmental stressors including cold, drought, soil pollution, and salinity (Ding et al., 2014; Garg, Chevala, Shankar, & Jain, 2015; Song et al., 2015; Yaish, Al‐Lawati, Al‐Harrasi, & Patankar, 2018).

Besides the reproducible epigenetic changes in specific epiloci, the rate of spontaneous epimutations throughout the epigenome might also be significantly affected by environmental stress (Johannes & Schmitz, 2019). For example, Jiang et al. found that spontaneous epigenetic changes increased 45% after 10 generations of cultivation under salt stress in *Arabidopsis thaliana* (Jiang et al., 2014). In contrast, Eichten and Springer failed to find spontaneous epigenetic changes in maize under abiotic stress, including heat, cold, and UV stress (Eichten & Springer, 2015). In previous study, we found a significant increase of epigenetic diversity in response to salinity stress in *Alternanthera philoxeroides*, an invasive clonal plant (Shi et al., 2018). As there were no consistent changes at specific epiloci across replicated individuals, the observed increase of epigenetic diversity was largely due to a higher rate of spontaneous epimutations under stress, which is consistent with the results from *A. thaliana*. Given the results among studies and/or species, however, there is no consensus on the effects of environmental stress on the rate of spontaneous epimutations in plants. Moreover, it is unknown whether environmentally induced changes in spontaneous epimutation are inheritable from generation to generation (Johannes & Schmitz, 2019). The stability of such environmentally induced epimutations is critical for their evolutionary potentials (Artemov et al., 2017). Specifically, if these epigenetic changes revert to the previous status immediately after the stress was eliminated, it would be better to classify them as some kind of molecular plasticity, which could be adaptive but have different evolutionary dynamics comparing to stable heritable epigenetic changes (Herrera, Medrano, & Bazaga, 2016; Johannes & Schmitz, 2019; Verhoeven & Preite, 2014).

*Thlaspi arvense* L., also known as field pennycress, is a member of Brassicaceae and widely distributed in temperate regions of the northern hemisphere (An, Zeng, Zhang, & Zhong, 2015; Best & Mcintyre, 1975; Warwick, Francis, & Susko, 2002). *T*. *arvense* is a diploid plant (2*n* = 2*x* = 14) with a genome size of 539Mb (Dorn, Fankhauser, Wyse, & Marks, 2015; Mulligan, 1957, 1960). Similar to other Brassicaceae relatives, there are two ecotypes within the species, as the consequence of *FLC* (*FLOWERING LOCUS C*) mutation, the early‐flowering ecotype does not need a vernalization process to initiate flowering, whereas the vernalization is necessary for the late‐flowering ecotype to initiate flowering (Dorn, Johnson, Daniels, Wyse, & Marks, 2018; McIntyre & Best, 1978). Traditionally, it has long been thought as a farm weed, which has attracted increasing interest as an oilseed crop for biofuel production and as a cover crop for reducing soil erosion (Dorn et al., 2015; Mcginn et al., 2019; Mitich, 1996; Sedbrook, Phippen, & Marks, 2014).

In this study, we investigated the effect of salinity stress on the rate of spontaneous epimutations of DNA methylation in pennycress, using methylation‐sensitive amplification polymorphism (MSAP) markers. The MSAP markers can recognize epigenetic variation of DNA methylation at relatively low cost, providing a useful tool to investigate the rate of spontaneous epimutations along the whole epigenome of many individuals within and among populations. MSAP markers do have some limitations, however, for example, the relative lower resolution and data quality comparing with Next‐Generation‐Sequencing methods. However, the lower cost of MSAP markers enables us to investigate the population epigenetic variation among many individuals, providing informative preliminary evidence for the effect of salinity stress on the rate of spontaneous epimutation. Specifically, we addressed the following questions: (a) whether salinity stress results in significantly higher levels of epigenetic diversity in *T*. *arvense* populations compared to the control groups. If so, (b) whether the epigenetic diversity can be maintained in offspring that are grown under nonstressed environments. In addition, we addressed (c) whether salinity stress affected the magnitude of phenotypic variation in functionally important traits, including plant height, seed number, and hundred‐seeds weight.

## 2 MATERIALS AND METHODS

### 2.1 Sample preparation

To determine the maximum salinity where *T*. *arvense* could grow and generate viable seeds, a series of salt concentration gradients of 0, 50, 75, 100, 150, and 200 mmol/L were set to water the plants every day. 75 mmol/L of salinity was the threshold where the *T*. *arvense* had showed significant phenotype differences compared with the ones which were watered with saltwater <75 mmol/L; plants watered with saltwater concentrations >75 mmol/L resulted in death before flowering or seed abortion. Thus, a concentration of 75 mmol/L was selected as the optimal salt exposure condition in the following experiments (Table S1).

The formal experiment lasted three generations. The original seeds were collected from a single plant of *T. arvense* in the field in Kunming (Yunnan Province, China) and used to develop the first generation of plants (G1). These plants in G1 are half‐siblings or nearly full‐siblings, which may share highly similar genetic backgrounds even for half‐siblings as our recent study revealed extremely low genetic diversity (He = 0.03) within the same population in Kunming (Guan et al., 2020, in press). Accordingly, we thought the plants in G1 shared a nearly homozygous genome, which could effectively avoid the differences in maternal effects and genetic backgrounds. Seeds were placed on moist filter papers, stratifying at 4°C for seven days, and then germinated at room temperature. Seedlings were transplanted in to pot soil (one seedling per pot) and then developed in the greenhouse. After three days, the transplanted seedlings were assigned into two groups with 10 seedlings in each: one was irrigated with saltwater every day (stressed, S) throughout their growing season, whereas the other one was irrigated with tap water (control check, CK). Since DNA methylation can be induced by environmental stimuli, but also differentiated between tissues and developmental stages, only the third and fourth true leaves were sampled when the plants started to flower and dried in silica gel for DNA extraction. While the seeds were matured, the plant height from soil to the inflorescence's apex was measured, the seed number for each plant was counted, and the hundred‐grain weight of each plant was also measured. Then, the seeds from the first generation were used to develop the second generation. The offspring (G2) of CK were kept to infiltrate with tap water (CK‐CK); however, the offspring of S were assigned into two groups again: one was infiltrated with tap water (S‐CK), and another was infiltrated with saltwater (S‐S). Then, in the third generation (G3), the seeds of CK‐CK, S‐CK, and S‐S were also used to generate new offspring, but all of them were infiltrated with tap water every day, and thus, a total of three groups were generated in the third generation, including CK‐CK‐CK, S‐CK‐CK, and S‐S‐CK, respectively. In this experiment, a total of eight groups including 80 individuals were assigned, and each group composed of ten replicates from a single genetic background. Except for the differences of salt exposure, the culture conditions, leaf collection for MSAP, and phenotype determination were identical across the three generations.

### 2.2 Molecular experiment

The dried leaves were used to extract genome DNA, following the manual of the TIANGAN Plant Genomic DNA kit, and DNA samples were tested in 1% of agarose electrophoresis. MSAP is a genotyping technique which was derived from amplified fragment length polymorphism (AFLP), using a pair of isoschizomers, commonly *Hpa*II and *Msp*I which recognize the same tetranucleotide sequence 5′‐CCGG‐3′ but differed in the sensibility to the methylation status at cytosine, to digest genome DNA parallelly. Among them, *Hpa*II is sensitive to the hemi‐ or full methylation at internal cytosine, whereas *Msp*I is sensitive to hemi‐methylation at external cytosine (Schulz, Eckstein, & Durka, 2013). We conducted the MSAP experiments following Gao, Geng, Li, Chen, and Yang (2010), and a total of 10 pairs of *EcoR*I/*Hpa*II and *EcoR*I/*Msp*I primer combinations were used in the final selective amplification, including AA/TTG, AA/TAC, AC/ATC, AC/ACT, AC/TCG, AC/TCT, AC/ACG, AG/TGC, AG/TAG, and AT/TCA. The amplified products were then separated on 6% sequencing gels and silver‐stained in 0.15% AgNO3 for 10 min and developed in 2% NaOH containing 2 ml of formalin.

### 2.3 Data scoring and analysis

Raw data of MSAP analysis were obtained by coding the presence and absence of bands as “1” and “0,” respectively. Here, four digestion results were included in the matrix: I) (1 1) indicated both *Hpa*II and *Msp*I could cleave; II) (0 1) indicated only *Msp*I could cleave; III) (1 0) indicated only *Hpa*II could cleave; and IV) (0 0) indicated neither *Hpa*II nor *Msp*I could cleave. To distinguish experimental errors and spontaneous mutations, we also ran 22 replicate samples (27.5% of total samples) for two times to estimate the scoring error rate. In the raw data matrix, the first two columns represented individual code and the group (or population) to which they belonged, respectively; and the first row represented the locus code, and the *Hpa*II and *Msp*I profiles at each locus were encoded in a single cell simultaneously, separating with a space. The raw data matrix containing the four methylation types was used to analyze the pattern of DNA methylation variation. First, we confirmed the consistent loci across the individuals within the CK group and then tracked them in the next two generations, seeking how many loci were changed under the salt exposure and to what extent the salt‐induced variation can be passed on to their offspring.

However, the type IV of (0 0) case could result from either methylation variation, or genetic variation. Referencing to the classification method of “mixed score 1,” we separated the raw data matrix into “unmethylation matrix” and “methylation matrix” to avoid the noise from genetic variation (Gao et al., 2010; Schulz et al., 2013). For the methylation matrix, the type I (1,1) and type IV (0,0) were transformed as “0,” whereas the type II (0,1) and type III (1,0) were transformed as “1”；and for the unmethylation matrix, only the type I (1,1) was transformed as “1,” and the other three were transformed as “0.” The methylation matrix was used to analyze epigenetic diversity, principal coordinate analysis (PCoA), and analysis of molecular variance (AMOVA). We discarded the monomorphic loci that vary in <5% of the samples in analyses of divergence.

The calculation of epigenetic diversity parameters including Shannon's diversity index (I), expected heterozygosity (H_e_), number of private bands (NPB), and percentage of polymorphic bands (PPB) was in GenAlEX 6.503 with default parameters (Peakall & Smouse, 2012). The PCoA and AMOVA were also performed in GenAlEX 6.503 with 9,999 permutations and defaulted other parameters.

In order to compare the effects induced by salt exposures across generations, we calculated the response ratio (value of treatment/ value of control) of Shannon's diversity index, plant height, seed number, and hundred‐grain weight, respectively. If the response ratio is significantly greater than 1, it indicates that the value of the treatment group is significantly higher than that of the control group, and vice versa. A larger response ratio in the second generation of treatment group (i.e., S‐S/CK‐CK) than that of the first generation (S/CK) suggests that the effect of salinity stress has become more prominent with time. For the response ratio of Shannon's diversity index as well as phenotypic traits (i.e., plant height, seed number, and hundred‐grain weight), we used a randomization procedure to produce replications and calculated standard error of mean (*SEM*) (Manly, 2007). The significance of raw phenotypic traits between generations was tested in GraphPad Prism 8.0.1 (www.graphpad.com) using an unpaired *t* test. Furthermore, the response ratio of coefficients of variation (CV) in plant height, seed number, and hundred‐grain weight were also calculated.

## 3 RESULTS

### 3.1 Epigenetic variation pattern

Our MSAP analysis showed that most of the sites remained unmethylated under both control and salt stress conditions; and salt stress reduced the number of methylation sites. 138 and 121 methylation sites were detected in the CK and S groups, accounting for 45.10% and 39.54% of the total sites, respectively. In the G1 generation, the number of methylation sites of the salt stress group also decreased compared with the control group. As the results show, there were 128 methylation sites in the CK‐CK group, accounting for 41.83% of the total sites, while only 110 methylation sites, accounting for 35.95% of the total sites, were detected in the S‐S group. Moreover, the number of methylation sites in the S‐S group was 5.88% lower than that in the CK‐CK group. Further analysis showed that the number of hypomethylation sites was higher than hypermethylation sites in the salt stress group compared with the control group of the same generation, which should have been caused by the hypomethylation of medial cytosine. This trend was more obvious in the S‐S group compared with the S group, which experienced two successive generations of salt stress. Even so, most of the methylation patterns were conservative and not affected by salt stress. The environmentally induced DNA methylation changes always involve the pathways or genes related to adaptation (Artemov et al., 2017; Banerjee & Roychoudhury, 2017). The salt‐induced DNA methylation variation in this study might also be related to adaptation.

**Figure 1:**
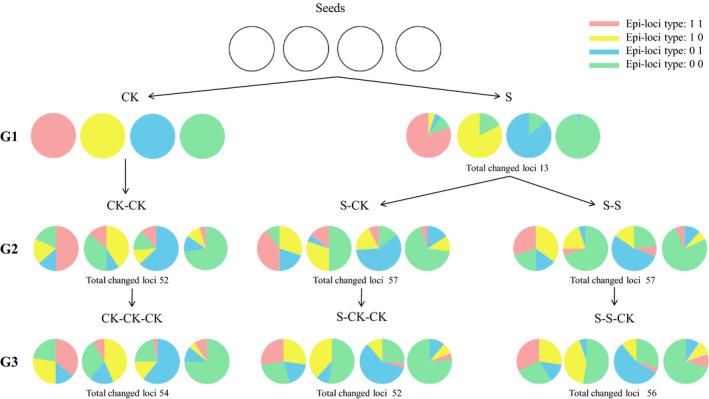
The dynamic changes of methylation status revealed by MSAP markers in three successive generations of pennycress growing under salinity stress and control environmental conditions. The four colors denote different methylation status (i.e., 0 1, 1 0, 1 1, and 0 0 in MSAP), and the four pie charts present the four epiloci groups with different methylation status in the CK group. The pie charts in the first row (G1 generation) shows higher levels of epigenetic diversity (i.e., the richness and evenness of color) in salinity groups (S) than those in the control check groups (CK). The pie charts in the left column suggest that the methylation status can change in three successive generations even under unstressed environments (e.g., CK vs. CK‐CK vs. CK‐CK‐CK)

We also found the percentage of the same varied methylation sites in different groups were 4.25%–18.62%. Among them, the S group had the lowest percentage with 13 methylation sites, and the S‐S and S‐CK groups had the highest percentage both with 57 sites. The methylation pattern of CK‐CK and CK‐CK‐CK groups without salt stress is conservative, but there were also some variations, which may have been caused by different culture conditions or random fluctuations between generations. Four types of methylation sites of the S group were conservative, which indicated that salt treatment on the first generation had little effect (Figure 1). In the second generations after salt stress, there were significant variations in S‐CK, S‐S, S‐CK‐CK, and S‐S‐CK groups. It is interesting that most of the methylation sites type (1 0) in the offspring of the S group changed to methylation sites type (0 0) (Figure 1).

### 3.2 Epigenetic diversity

In this study, an MSAP experiment was conducted using 80 individuals of eight experimental groups and 306 distinct loci were amplified, among them 4.82% of them were polymorphic, and only a 2.2% scoring error rate was detected in the two‐time analyses. The results showed that all the experimental groups had lower epigenetic diversity compared to castor bean (He, Xu, Li, Wang, & Liu, 2017). The average Shannon diversity index of eight groups was 0.025; the average He was 0.016, and the average PPB was 4.82% (Table 1). The highest epigenetic diversity was found in the S‐S group (I = 0.037, H_e_ = 0.024), which endured two successive generations of salt stress. The PPB was 6.86%, of which six loci were unique to this group. In contrast, the CK‐CK‐CK group showed the lowest Shannon diversity index (0.013), He (0.009), and PPB (2.94%) values. Moreover, one specific band was detected in S, CK‐CK, and S‐CK groups, respectively.

**Table 1:** Epigenetic diversity among the eight experimental groups

Pop	*N*	I	He	PPB (%)	NPB
CK	10.000	0.017	0.012	2.94	8
S	10.000	0.021	0.014	4.58	1
CK‐CK	10.000	0.017	0.010	3.92	1
S‐CK	10.000	0.029	0.019	6.21	1
S‐S	10.000	0.037	0.024	6.86	6
CK‐CK‐CK	10.000	0.013	0.009	2.94	5
S‐CK‐CK	10.000	0.025	0.017	4.58	3
S‐S‐CK	10.000	0.038	0.026	6.54	5
Mean	10.000	0.025	0.016	4.82	–

Note

Pop, name of experimental group; *N*, sample size in each group; I, Shannon's diversity index; He, expected heterozygosity; NPB, number of private bands; PPB, percentage of polymorphic bands.

**Figure 2:**
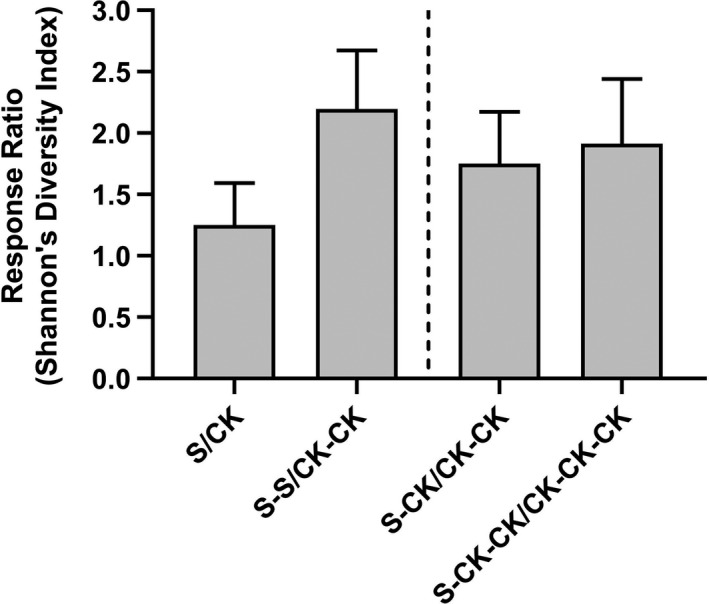
The response ratio of Shannon's diversity index of epiloci (i.e., epigenetic diversity) for plants grown under salinity stress and control environmental conditions. The response ratio of Shannon's diversity index was calculated as the value of treatment group/ value of control group. The treatment groups are scaled down according to the control group of the same generation. The standard error of mean was obtained using a randomization procedure (see details in M & M)

After the first generation of salt stress, the epigenetic diversity of the S group was increased compared with that of CK (Figure 2). Furthermore, the epigenetic diversity of the S‐S group under two successive generations of salt stress was further increased compared with the S group. Similarly, the epigenetic diversity of the S‐CK group under salt stress was higher than that of the CK‐CK groups, and this trend still existed in the S‐CK‐CK group, which was eliminated from salt stress for two generations (Figure 2).

### 3.3 Epigenetic differentiation and heritability

**Figure 3:**
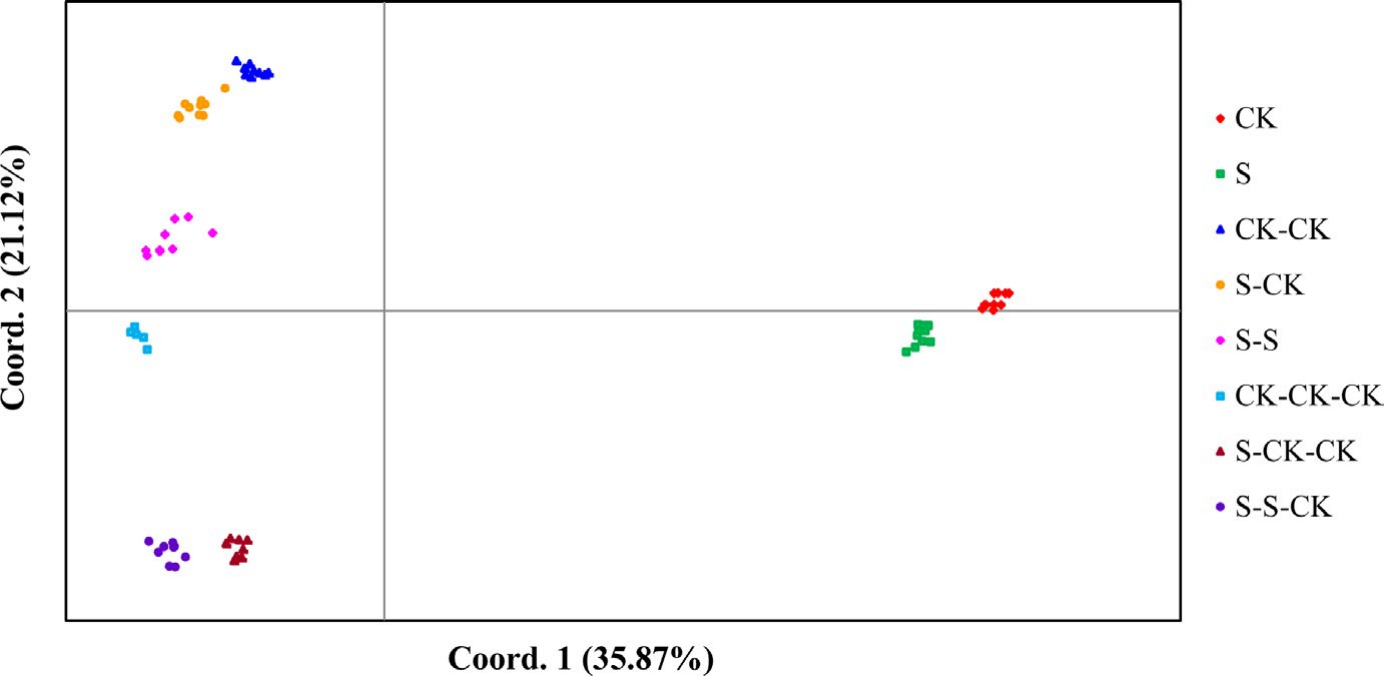
The principal coordinates analysis (PCoA) of eight experimental groups

Analysis of molecular variance (AMOVA) and principal coordinates (PCoA) showed that there was significant epigenetic differentiation among the eight experimental groups (Table 2, Figure 3). About 91% of the epigenetic variation was found between the experimental groups, whereas only 9% of the variation existed within the group (*p* < .001). When the cultivated conditions of parents and offspring were changed or not, there was no significant difference in the percent of stable epigenetic loci. For example, under the same cultivated condition, 61.76% to 78.43% of the loci were stably inherited. Similarly, when the cultivation environment was changed, 63.07% to 74.51% of the loci were stably inherited (Table 3).

**Table 2:** Analysis of molecular variance (AMOVA) for the experimental groups

Source	*df*	SS	%	*p* (rand>= data)
Among groups	7	1971.775	91	.000
Within groups	72	203.200	9	
Total	79	2,174.975	100	

**Table 3:** The methylation variation pattern between different experimental groups

	Variation site (%)	Conservative sites (%)	Total sites
Environment constant			
CK to CK‐CK	112 (36.60%）	194 (63.40%）	306
CK‐CK to CK‐CK‐CK	66 (21.57%）	240 (78.43%）	306
S to S‐S	117 (38.24%）	189 (61.76%）	306
Environment changed			
S to S‐CK	113 (36.93%）	193 (63.07%）	306
S‐CK to S‐CK‐CK	84 (27.45%）	222 (72.55%）	306
S‐S to S‐S‐CK	78 (25.49%）	228 (74.51%）	306

### 3.4 Phenotypic variation

**Figure 4:**
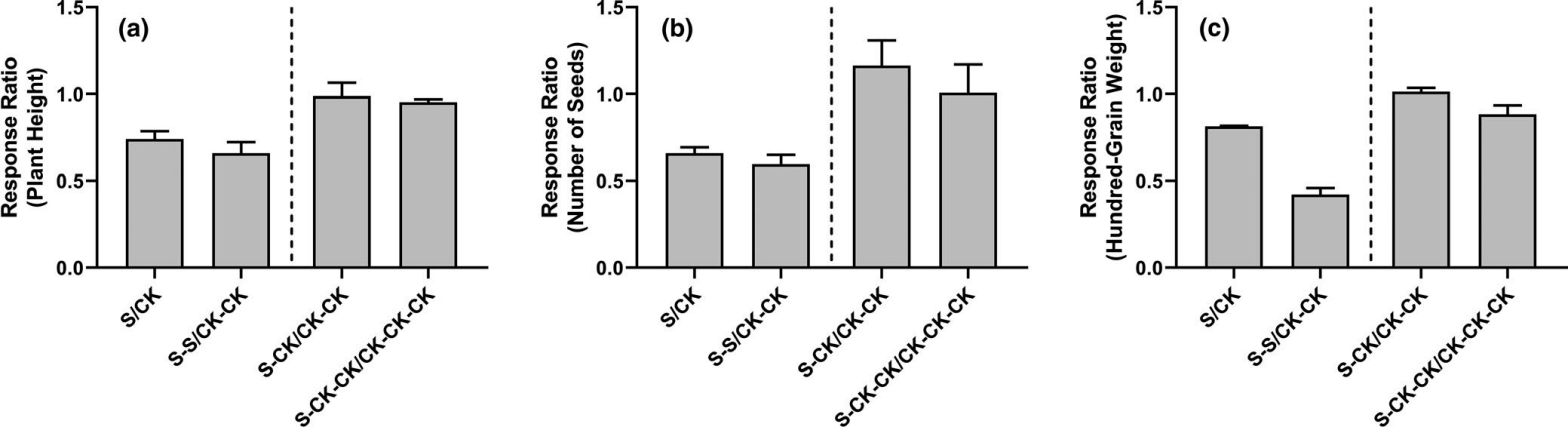
The response ratio of plant height (a), seed number (b), and hundred‐grain weight (c) for plants grown under salinity stress and control environmental conditions. The response ratio was calculated as the value of treatment group/ value of control group. The treatment groups are scaled down according to the control group of the same generation. The standard error was obtained using a randomization procedure (see details in Section 2)

As predicted, salt stress caused a significant decrease in the plant height of the S group compared with the CK group in the G1 generation (*p* < .0001), as well as in the S‐S group compared with CK‐CK group (*p* < .001) in the G2 generation (Figure 4, Figure S1). After the salt stress was eliminated in the next generation, plant height recovery reached the same level as the control in the same generation. This trend was also found in seed number and hundred‐grain weight (Figure 4).

**Figure 5:**
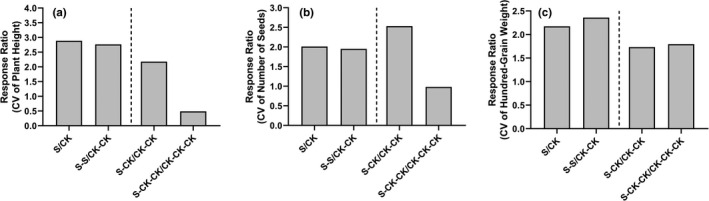
The response ratio of phenotypic coefficient of variation (standard deviation/ mean) of plant height (a), seed number (b), and hundred‐rain weight (c) for plants grown under salinity stress and control environmental conditions. The variation coefficient of response ratio was calculated as the value of treatment group/ value of control group. The treatment groups are scaled down according to the control group of the same generation. As CV is a parameter of a group rather than an individual plant, there are no replicates at group level in this study. And no error bars are available here

According to the phenotypic coefficient of variation (standard deviation/ mean) in pennycress (Figure 5), we found that under salt stress, the plant height, seed number, and hundred‐grain weight were all highly variable compared with control in the same generation. However, when salt stress was eliminated in the next generation, the variation of plant height caused by salt stress decreased (i.e., S‐CK/CK‐CK < S/CK) and was even lower than the control group after two generations (i.e., S‐CK‐CK/ CK‐CK‐CK < S‐CK/CK‐CK). Unlike the plant height, the variation of number of seeds in S‐CK‐CK group was nearly the same as that of the CK‐CK‐CK group, and hundred‐grain weight still varied even though two generations of recovery in the S‐CK‐CK group, compared with the CK‐CK‐CK group. It is interesting that the S‐CK group produced slightly more seeds than the CK‐CK group, but this effect was not statistically significant. This might be caused by the compensative reproduction of offspring, that is, the transgenerational effects of the parental exposure to salt, or just an artifact due to random environmental fluctuation or a sampling effect. More data are needed to separate the two competing hypotheses.

## 4 DISCUSSION

Recent studies have demonstrated that epigenetic variation was affected by environmental stimulus, genetic variation, and geographic origin together (Artemov et al., 2017; Herman & Sultan, 2016; Kawakatsu et al., 2016; Preite et al., 2018). Thus, it was a good choice to use nearly isogenic plants to study the effects of environmental stimulus on epigenetic variation. In this study, we investigated the effect of salinity stress on the epigenetic variation of DNA methylation in field pennycress using MSAP markers. We found that (a) the population epigenetic diversity increased in response to the stimulation of salinity stress; and (b) this “stimulation effect” could be transferred partially as stress memory to offspring in nonstressed environment; (c) we did observe parallel trends in phenotypic variation, that is, the coefficient of variation in a few functionally important traits (plant height, seed number, and hundred‐grain weight) was higher in plants grown under salinity stress compared with control groups. And previous studies in closely related species *A*. *thaliana* also demonstrated that the changes both in phenotypes and DNA methylation under salt stresses could maintain in their off‐springs (Bilichak, Ilnystkyy, Hollunder, & Kovalchuk, 2012; Groot et al., 2016).

The most prominent finding in our study is the higher levels of epigenetic diversity in salinity stress groups than their control groups, respectively, that is, S versus. CK, S‐S versus CK‐CK (Figure 2). This pattern is consistent with our previous finding in *Alternanthera philoxides*, a clonal perennial weed (Shi et al., 2018). Given the contrasting habit and evolutionary history of these two species (e.g., annual vs. perennial, seed vs. clonal), it is reasonable to hypothesize that the elevated trend of epigenetic diversity in response to salinity stress is also held in other plant species, which thus deserve more experimental investigations. Theoretically, the higher levels of epigenetic diversity could be explained by a higher rate of stochastic epimutation under stress. Previous study found that spontaneous epimutation was significantly higher under salinity stress, which could be due to a higher error rate on enzyme systems under stress (Jiang et al., 2014). In this study, we found obvious spontaneous epimutation in pennycress even in nonstressed environments (e.g., the control group). Moreover, the stimulation effect of salinity stress increased with the stress time duration. Further research on enzyme activities, involving methylation and demethylation processes, could be examined in the future to test this hypothesis. In this study, the epigenetic changes were also found between generations and might have effects on the comparison of epigenetic inheritability; however, our conclusion was valid that the epigenetic diversity within the salt‐stressed groups was higher than that of nonstressed groups. Furthermore, the PCoA also revealed that the individual differences within the salt‐stressed groups were elevated, compared with the nonstressed groups, which also supported our conclusion.

In contrast, we did not find any predictable epigenetic change on specific epiloci across replicated individuals in response to salinity stress. Similar results were also reported in *Taraxacum officinale* in response to drought and salicylic acid treatments (Preite et al., 2018). It has been suggested that epigenetic modification plays an important role in the adaptive response of plants, including priming or acclimation to environmental stress. Such epiloci usually are associated with specific epiloci (CHH or CHG) in functional regions such as promoters. Due to the limitation of MSAP markers, we cannot separate the CG sites from other sites. If the CG sites were over‐represented in our study, it would not be surprising that most epiloci are not specifically responsive to environmental stress.

In this study, the environmentally induced epigenetic diversity was to some degree maintained in the next generation (G2), suggesting the parent environmental stress memory and the direct stress could affect the embryo in the seeds, which is on the mother plants. However, the effect of environmental stress on epigenetic diversity was still obvious in the G3 generation, suggesting that the direct stress on the embryo of seeds was not the sole factor, and that stress memory is also involved. At locus level, we also found that nearly 60% of the total epiloci could be transferred to offspring both under constant and variable environment conditions. Thus, changes to enzyme maintenance systems could be responsible for the inheritable epiloci across generations.

It is unknown how spontaneous epimutation can affect phenotypes. Current evidence suggests that at least some of epimutations can have significant impacts on functionally important traits (Alvarado, Rajakumar, Abouheif, & Szyf, 2015; Johannes et al., 2009; Kooke et al., 2015; Schmid et al., 2018). In this study, we did find a parallel trend of increased variation in a few functionary traits like plant height, seed quality, and seed numbers. The plants used in this study shared the same genotype as they are derived from one family of a selfing plant species. However, they showed higher levels of phenotypic variation under environmental stress than control groups. This variation could not be explained by genetic variation or heterogenous microenvironment. Instead, increased epigenetic variation might be partially responsible for this phenotypic variation. More rigorous experiments are needed to confirm the association between spontaneous epimutation and phenotypic variation.

The major limitations of this study were associated with the MSAP markers. Specifically, as anonymous makers, MSAP cannot provide information on sequence annotation (e.g., gene body methylation, methylation at repeat sequence or promoter regions), which are critical for the functional significance of DNA methylation. In addition, just like AFLP markers, most of the MSAP markers could be functionally neutral and thus the epigenetic diversity might be not straightforwardly linked with phenotypic variations. In future work, methylation‐sensitive restriction enzymes for genotyping by sequencing (GBS) could be used to provide more information than MSAP. And more powerful tools including epigenome, transcriptome, and mutation accumulation lines could be included in experimental design to shed light on the mechanisms and evolutionary consequences of spontaneous epimutations in response to stress in plants.

## 5 DATA ACCESSIBILITY STATEMENT

Epigenetic data of eight groups are available through documents in the Dryad repository at https://doi.org/10.5061/dryad.kprr4xh2w.

## CONFLICT OF INTEREST

The authors declare no competing financial interests.

## AUTHOR CONTRIBUTION

**Yupeng Geng:** Data curation (equal); Funding acquisition (equal); Supervision (equal); Writing‐original draft (equal). **Na Chang:** Data curation (equal); Investigation (equal); Resources (equal). **Yuewan Zhao:** Investigation (equal); Resources (equal). **Xiaoying Qin:** Investigation (equal); Resources (equal). **Shugang Lu:** Investigation (equal); Resources (equal). **James Crabbe:** Writing‐original draft (equal); Writing‐review & editing (equal). **Yabin Guan:** Data curation (equal); Investigation (equal); Resources (equal); Writing‐original draft (equal). **Ticao Zhang:** Funding acquisition (equal); Resources (equal); Supervision (equal); Writing‐original draft (equal).

## Supporting information

Supplementary Material
