# Supplementary material for: Increased epigenetic diversity and transient epigenetic memory in response to salinity stress in Thlaspi arvense
Source: Ecol Evol. 2020 Sep 20;10(20):11622–30. doi: 10.1002/ece3.6795 (PMC7593180; doi:10.1002/ece3.6795)
Supplement: Supplementary file 1 — Supplementary Material [file ECE3-10-11622-s001.docx]

Figure S1 The plot of raw phenotypic traits of plant height (a), seed number (b), hundred-grain weight (c) for plants grown under salinity stress and control environmental conditions. Values represent means ± SEM.


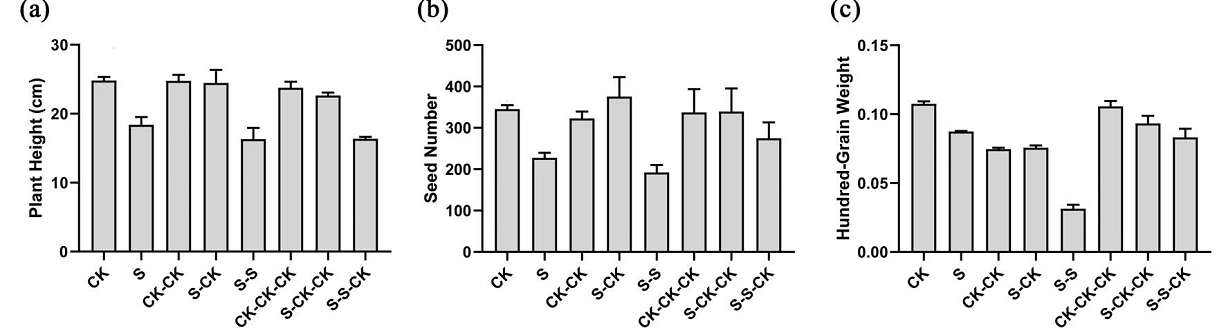


Table S1 Preliminary test for determining the optimum salt concentration in salt stress.

| Salt Concentration (mmol/L) | | n | NDF | NDM | NM |
| --- | --- | --- | --- | --- | --- |
| 0 | 6 | 0 | 0 | 6 |  |
| 50 | 6 | 0 | 0 | 6 |  |
| 75 | 6 | 0 | 0 | 6 |  |
| 100 | 6 | 1 | 2 | 3 |  |
| 150 | 6 | 2 | 3 | 1 |  |
| 200 | 6 | 6 | 0 | 0 |  |

N, number of replicates in each salt concentration; NDF, number of plants died before flowering; NDM, number of plants died before maturity; NM, number of mature plants.
